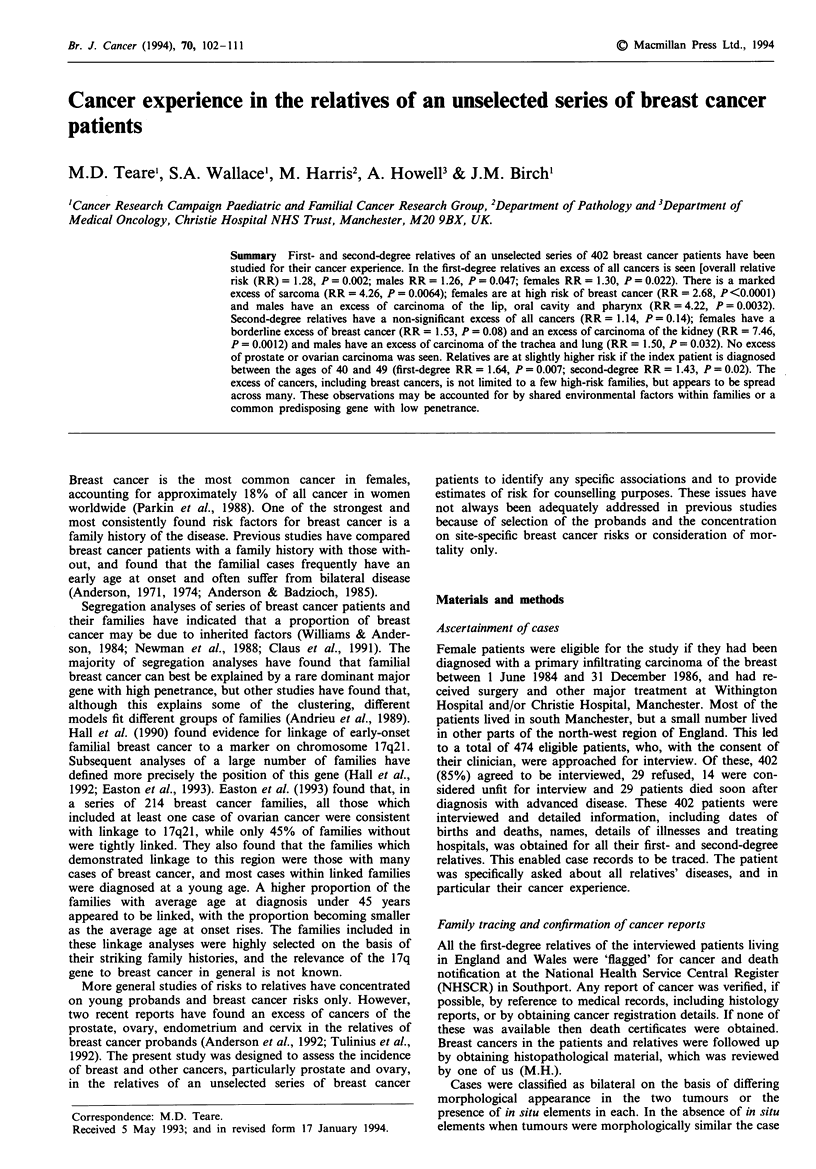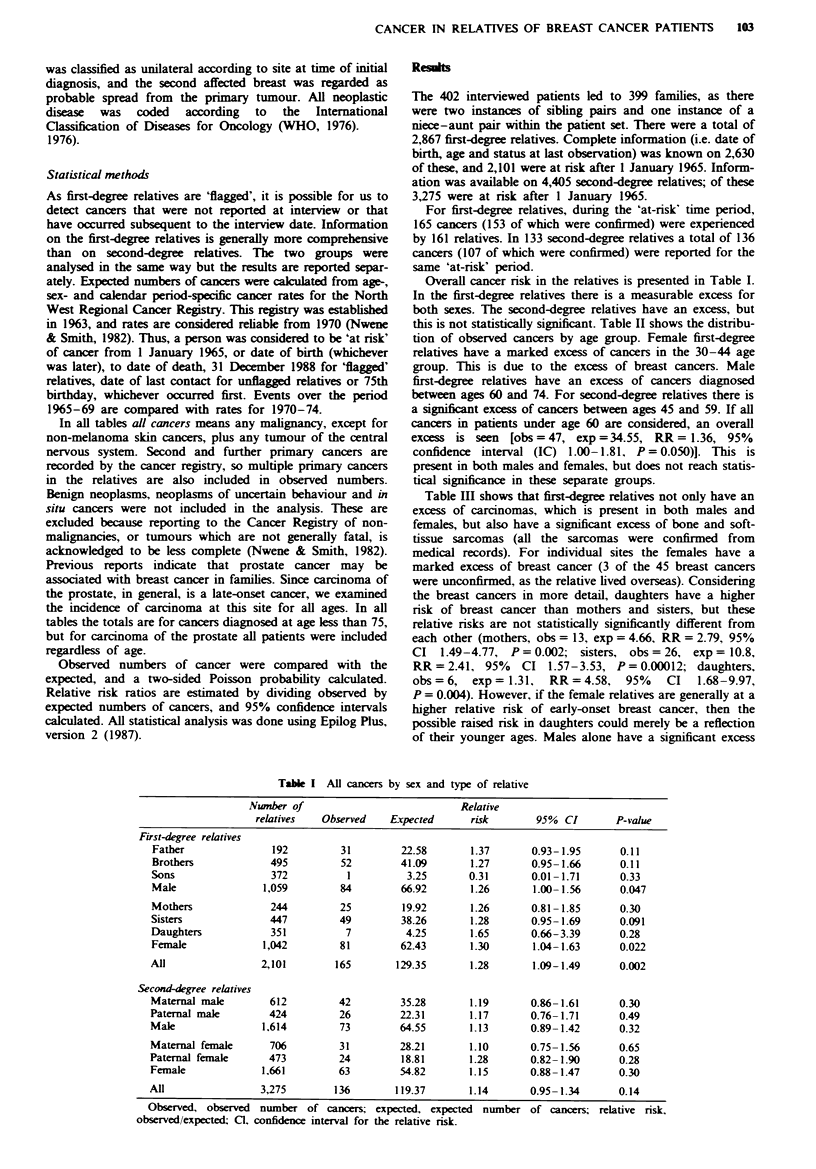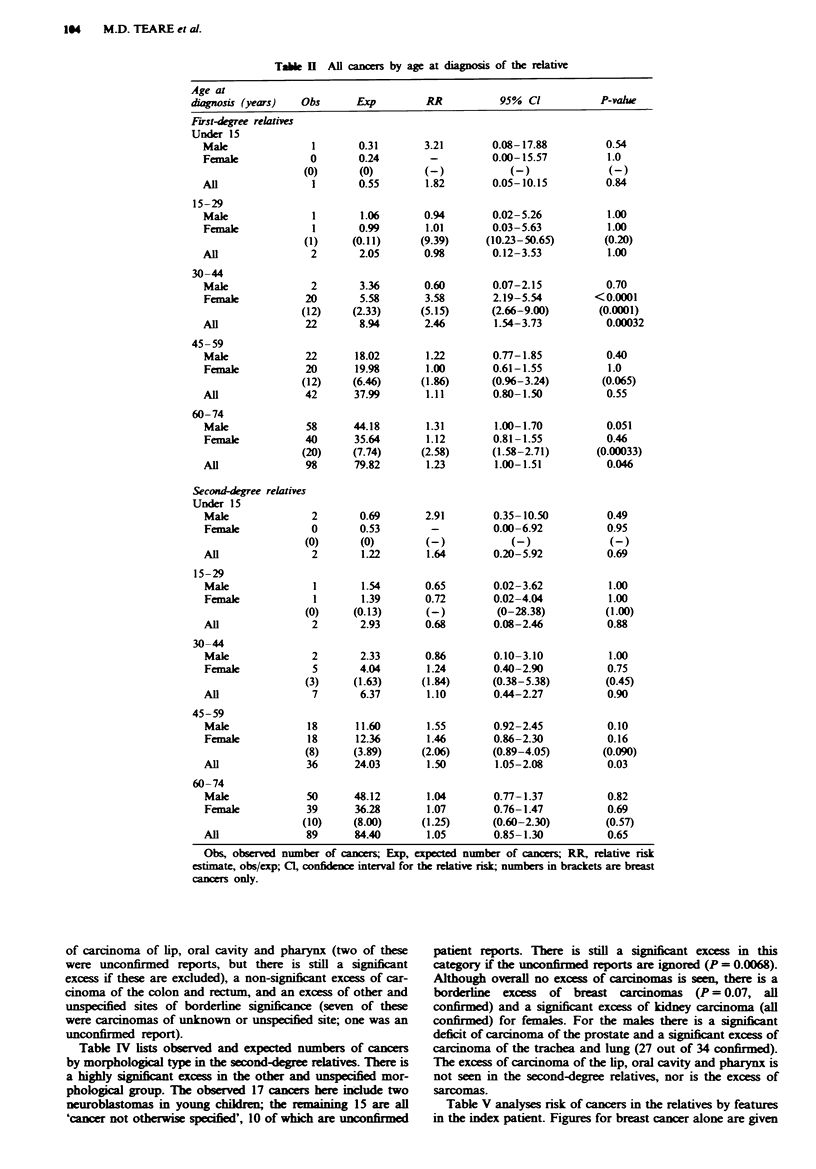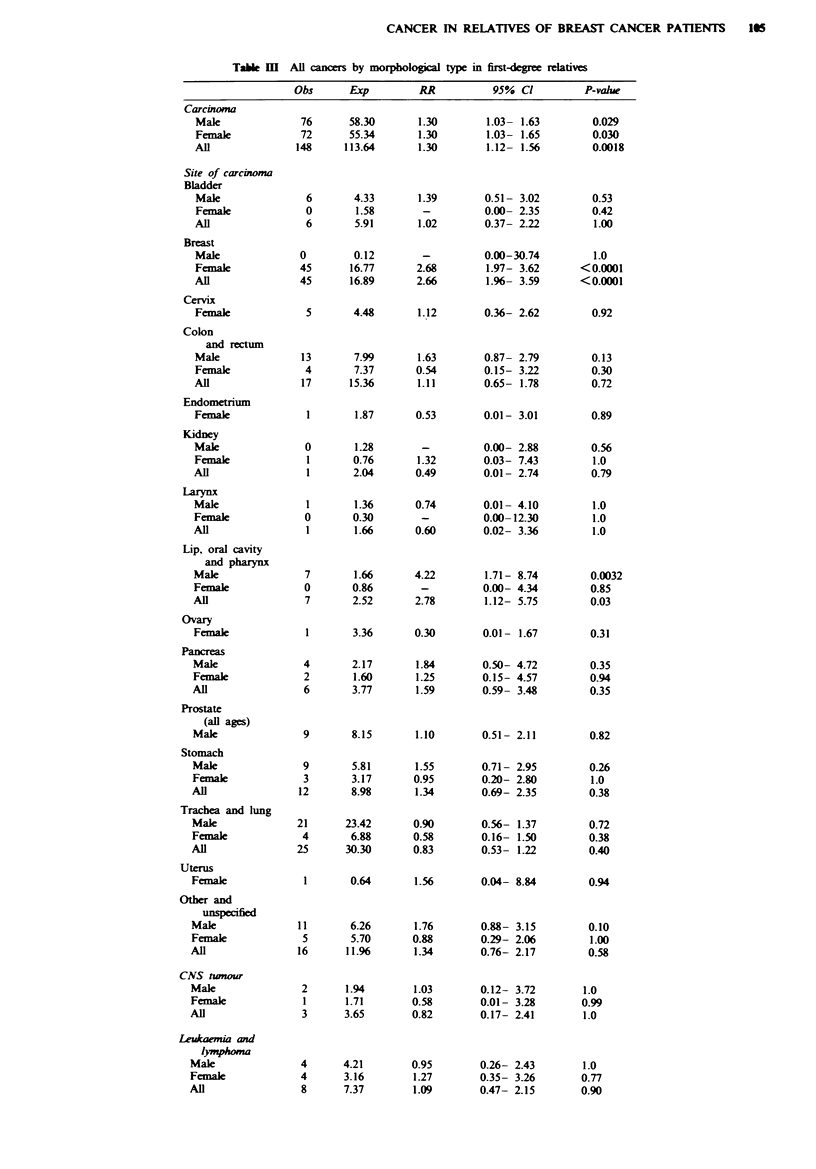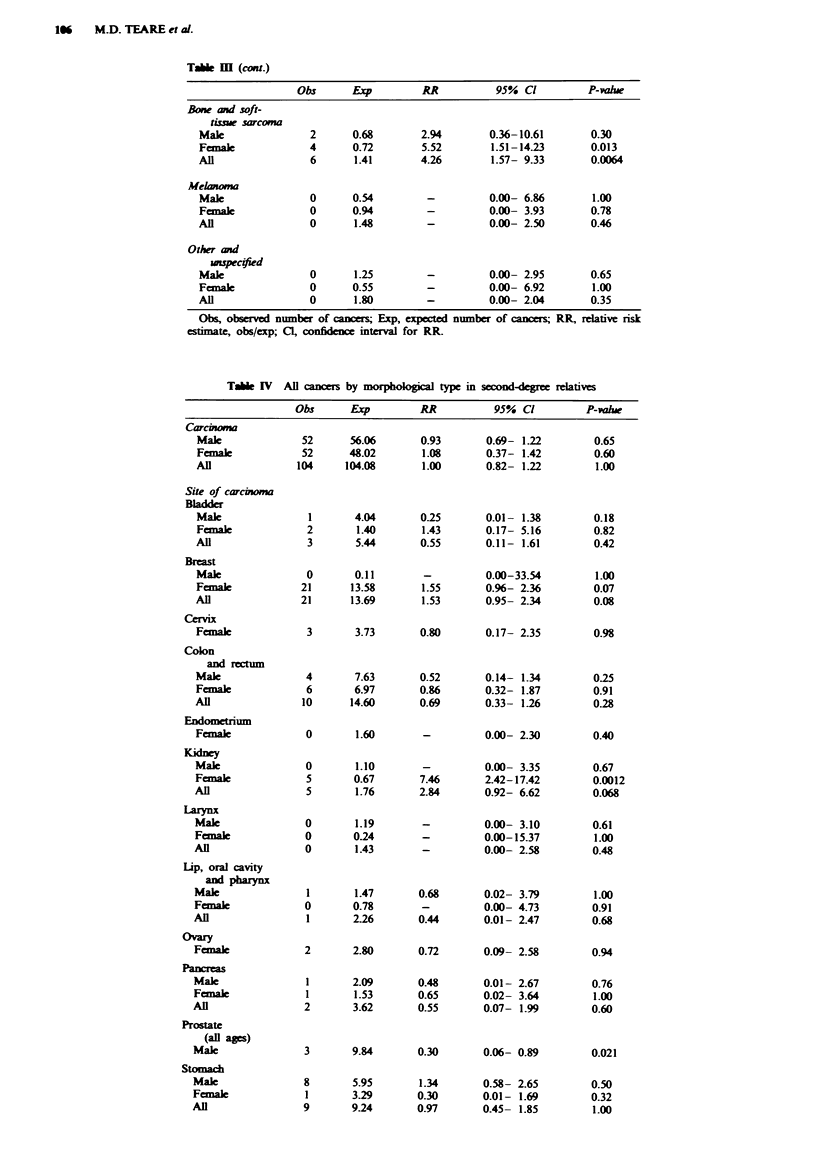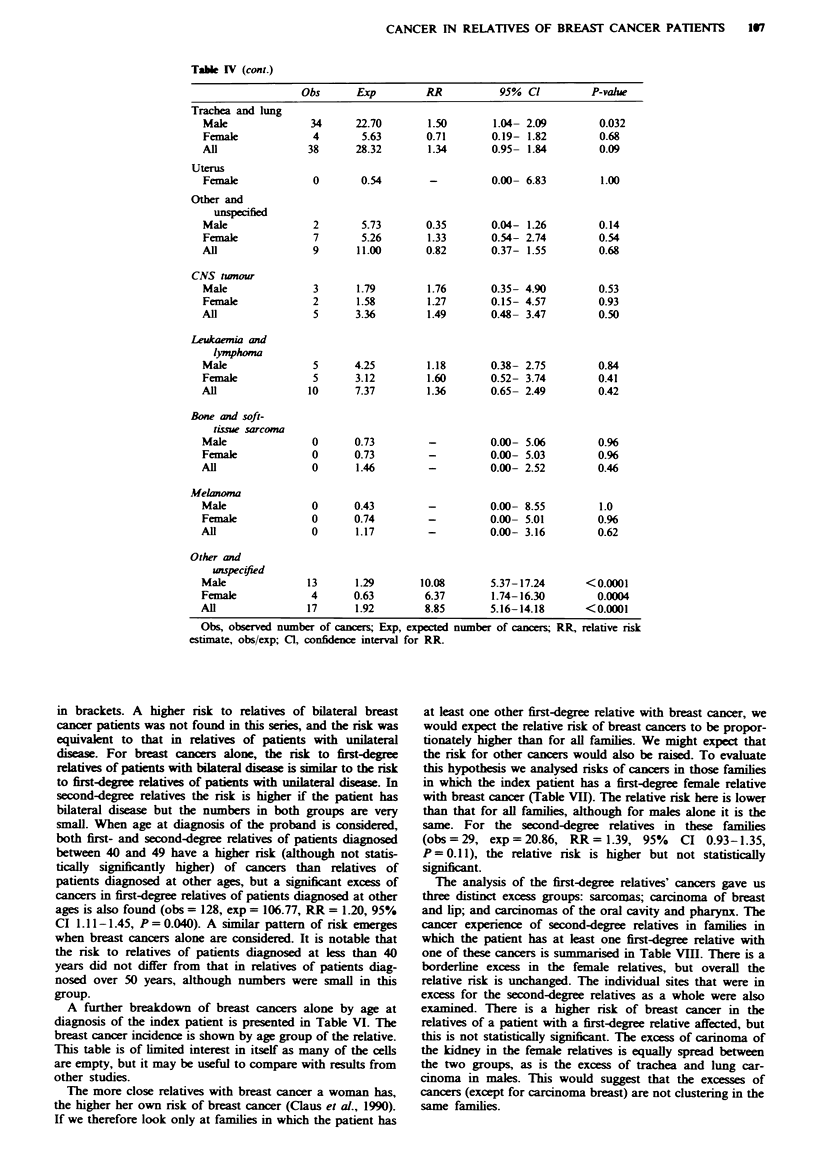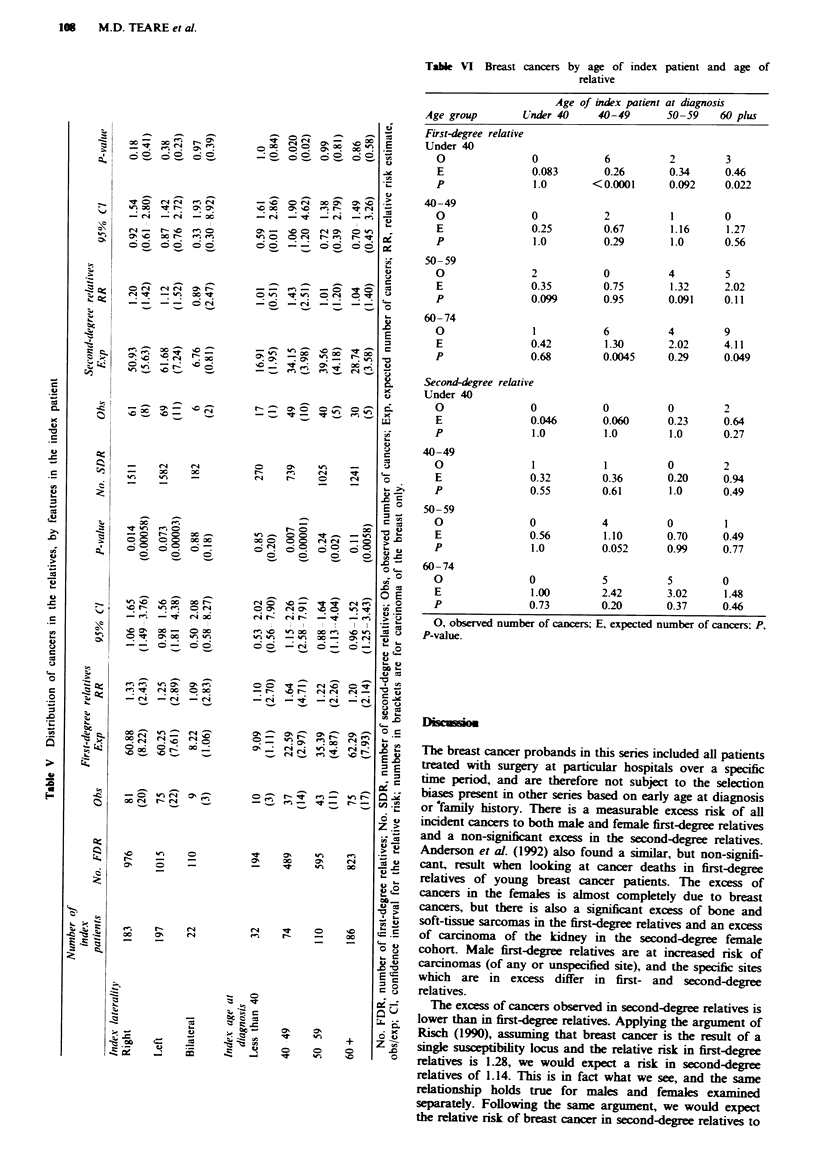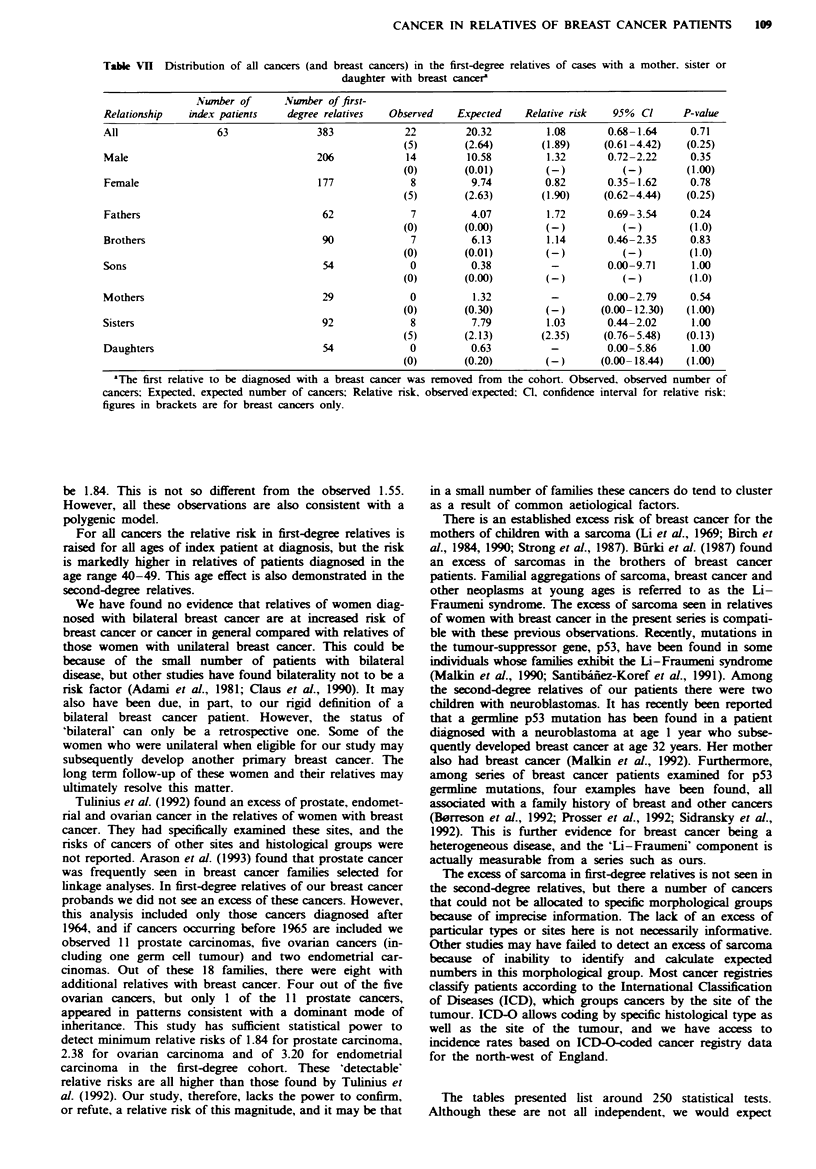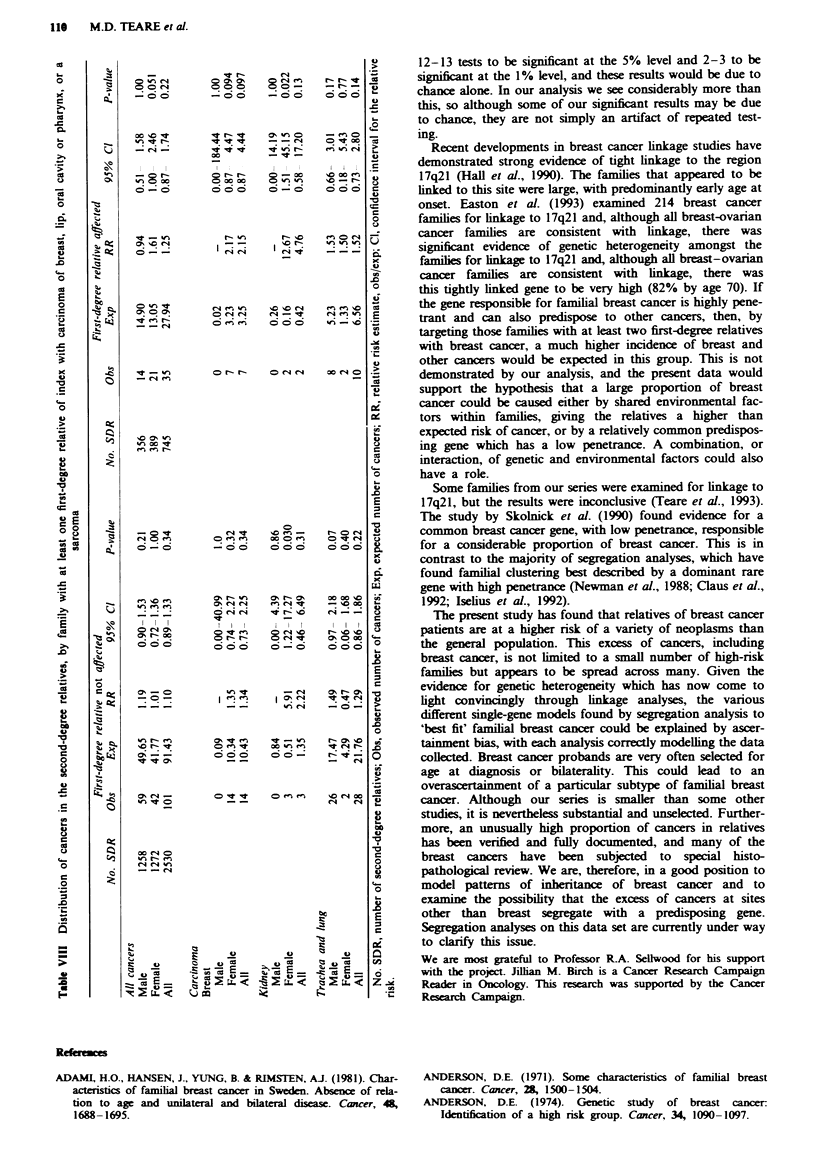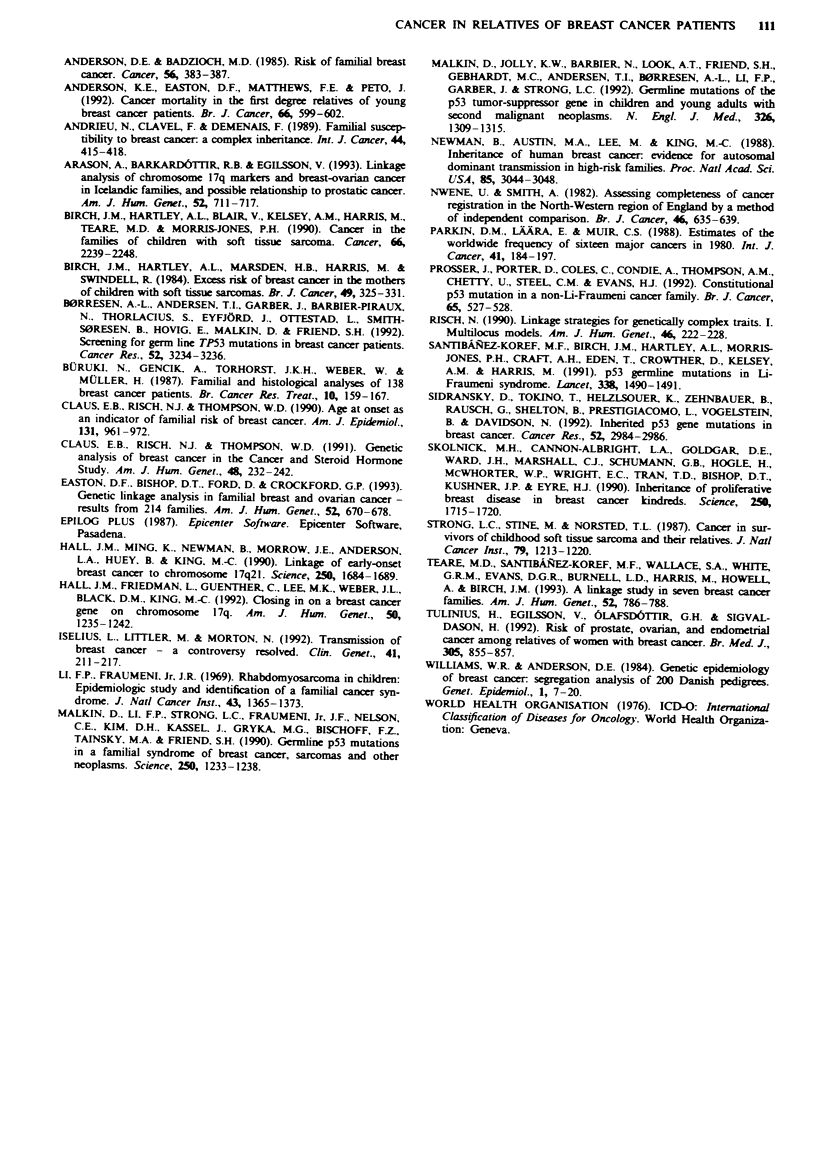# Cancer experience in the relatives of an unselected series of breast cancer patients.

**DOI:** 10.1038/bjc.1994.257

**Published:** 1994-07

**Authors:** M. D. Teare, S. A. Wallace, M. Harris, A. Howell, J. M. Birch

**Affiliations:** Cancer Research Campaign Paediatric and Familial Cancer Research Group, Christie Hospital NHS Trust, Manchester, UK.

## Abstract

First- and second-degree relatives of an unselected series of 402 breast cancer patients have been studied for their cancer experience. In the first-degree relatives an excess of all cancers is seen [overall relative risk (RR) = 1.28, P = 0.002; males RR = 1.26, P = 0.047; females RR = 1.30, P = 0.022). There is a marked excess of sarcoma (RR = 4.26, P = 0.0064); females are at high risk of breast cancer (RR = 2.68, P < 0.0001) and males have an excess of carcinoma of the lip, oral cavity and pharynx (RR = 4.22, P = 0.0032). Second-degree relatives have a non-significant excess of all cancers (RR = 1.14, P = 0.14); females have a borderline excess of breast cancer (RR = 1.53, P = 0.08) and an excess of carcinoma of the kidney (RR = 7.46, P = 0.0012) and males have an excess of carcinoma of the trachea and lung (RR = 1.50, P = 0.032). No excess of prostate or ovarian carcinoma was seen. Relatives are at slightly higher risk if the index patient is diagnosed between the ages of 40 and 49 (first-degree RR = 1.64, P = 0.007; second-degree RR = 1.43, P = 0.02). The excess of cancers, including breast cancers, is not limited to a few high-risk families, but appears to be spread across many. These observations may be accounted for by shared environmental factors within families or a common predisposing gene with low penetrance.


Br. J. Cancer (1994), 70, 102–111 © Macmillan Press Ltd., 1994

### Cancer experience in the relatives of an unselected series of breast cancer patients

M.D. Teare[1], S.A. Wallace[1], M. Harris[2], A. Howell[3] & J.M. Birch[1]

[1]*Cancer Research Campaign Paediatric and Familial Cancer Research Group,* [2]*Department of Pathology and* [3]*Department of Medical Oncology, Christie Hospital NHS Trust, Manchester, M20 9BX, UK.*

**Summary** First- and second-degree relatives of an unselected series of 402 breast cancer patients have been studied for their cancer experience. In the first-degree relatives an excess of all cancers is seen [overall relative risk (RR) = 1.28, $P = 0.002$; males RR = 1.26, $P = 0.047$; females RR = 1.30, $P = 0.022$). There is a marked excess of sarcoma (RR = 4.26, $P = 0.0064$); females are at high risk of breast cancer (RR = 2.68, $P < 0.0001$) and males have an excess of carcinoma of the lip, oral cavity and pharynx (RR = 4.22, $P = 0.0032$). Second-degree relatives have a non-significant excess of all cancers (RR = 1.14, $P = 0.14$); females have a borderline excess of breast cancer (RR = 1.53, $P = 0.08$) and an excess of carcinoma of the kidney (RR = 7.46, $P = 0.0012$) and males have an excess of carcinoma of the trachea and lung (RR = 1.50, $P = 0.032$). No excess of prostate or ovarian carcinoma was seen. Relatives are at slightly higher risk if the index patient is diagnosed between the ages of 40 and 49 (first-degree RR = 1.64, $P = 0.007$; second-degree RR = 1.43, $P = 0.02$). The excess of cancers, including breast cancers, is not limited to a few high-risk families, but appears to be spread across many. These observations may be accounted for by shared environmental factors within families or a common predisposing gene with low penetrance.

Breast cancer is the most common cancer in females, accounting for approximately 18% of all cancer in women worldwide (Parkin *et al.*, 1988). One of the strongest and most consistently found risk factors for breast cancer is a family history of the disease. Previous studies have compared breast cancer patients with a family history with those without, and found that the familial cases frequently have an early age at onset and often suffer from bilateral disease (Anderson, 1971, 1974; Anderson & Badzioch, 1985).

Segregation analyses of series of breast cancer patients and their families have indicated that a proportion of breast cancer may be due to inherited factors (Williams & Anderson, 1984; Newman *et al.*, 1988; Claus *et al.*, 1991). The majority of segregation analyses have found that familial breast cancer can best be explained by a rare dominant major gene with high penetrance, but other studies have found that, although this explains some of the clustering, different models fit different groups of families (Andrieu *et al.*, 1989). Hall *et al.* (1990) found evidence for linkage of early-onset familial breast cancer to a marker on chromosome 17q21. Subsequent analyses of a large number of families have defined more precisely the position of this gene (Hall *et al.*, 1992; Easton *et al.*, 1993). Easton *et al.* (1993) found that, in a series of 214 breast cancer families, all those which included at least one case of ovarian cancer were consistent with linkage to 17q21, while only 45% of families without were tightly linked. They also found that the families which demonstrated linkage to this region were those with many cases of breast cancer, and most cases within linked families were diagnosed at a young age. A higher proportion of the families with average age at diagnosis under 45 years appeared to be linked, with the proportion becoming smaller as the average age at onset rises. The families included in these linkage analyses were highly selected on the basis of their striking family histories, and the relevance of the 17q gene to breast cancer in general is not known.

More general studies of risks to relatives have concentrated on young probands and breast cancer risks only. However, two recent reports have found an excess of cancers of the prostate, ovary, endometrium and cervix in the relatives of breast cancer probands (Anderson *et al.*, 1992; Tulinius *et al.*, 1992). The present study was designed to assess the incidence of breast and other cancers, particularly prostate and ovary, in the relatives of an unselected series of breast cancer patients to identify any specific associations and to provide estimates of risk for counselling purposes. These issues have not always been adequately addressed in previous studies because of selection of the probands and the concentration on site-specific breast cancer risks or consideration of mortality only.

Correspondence: M.D. Teare.
Received 5 May 1993; and in revised form 17 January 1994.

### Materials and methods

### *Ascertainment of cases*

Female patients were eligible for the study if they had been diagnosed with a primary infiltrating carcinoma of the breast between 1 June 1984 and 31 December 1986, and had received surgery and other major treatment at Withington Hospital and/or Christie Hospital, Manchester. Most of the patients lived in south Manchester, but a small number lived in other parts of the north-west region of England. This led to a total of 474 eligible patients, who, with the consent of their clinician, were approached for interview. Of these, 402 (85%) agreed to be interviewed, 29 refused, 14 were considered unfit for interview and 29 patients died soon after diagnosis with advanced disease. These 402 patients were interviewed and detailed information, including dates of births and deaths, names, details of illnesses and treating hospitals, was obtained for all their first- and second-degree relatives. This enabled case records to be traced. The patient was specifically asked about all relatives' diseases, and in particular their cancer experience.

### *Family tracing and confirmation of cancer reports*

All the first-degree relatives of the interviewed patients living in England and Wales were 'flagged' for cancer and death notification at the National Health Service Central Register (NHSCR) in Southport. Any report of cancer was verified, if possible, by reference to medical records, including histology reports, or by obtaining cancer registration details. If none of these was available then death certificates were obtained. Breast cancers in the patients and relatives were followed up by obtaining histopathological material, which was reviewed by one of us (M.H.).

Cases were classified as bilateral on the basis of differing morphological appearance in the two tumours or the presence of *in situ* elements in each. In the absence of *in situ* elements when tumours were morphologically similar the case was classified as unilateral according to site at time of initial diagnosis, and the second affected breast was regarded as probable spread from the primary tumour. All neoplastic disease was coded according to the International Classification of Diseases for Oncology (WHO, 1976). 1976).

*Statistical methods*

As first-degree relatives are 'flagged', it is possible for us to detect cancers that were not reported at interview or that have occurred subsequent to the interview date. Information on the first-degree relatives is generally more comprehensive than on second-degree relatives. The two groups were analysed in the same way but the results are reported separately. Expected numbers of cancers were calculated from age-, sex- and calendar period-specific cancer rates for the North West Regional Cancer Registry. This registry was established in 1963, and rates are considered reliable from 1970 (Nwene & Smith, 1982). Thus, a person was considered to be 'at risk' of cancer from 1 January 1965, or date of birth (whichever was later), to date of death, 31 December 1988 for 'flagged' relatives, date of last contact for unflagged relatives or 75th birthday, whichever occurred first. Events over the period 1965–69 are compared with rates for 1970–74.

In all tables *all cancers* means any malignancy, except for non-melanoma skin cancers, plus any tumour of the central nervous system. Second and further primary cancers are recorded by the cancer registry, so multiple primary cancers in the relatives are also included in observed numbers. Benign neoplasms, neoplasms of uncertain behaviour and *in situ* cancers were not included in the analysis. These are excluded because reporting to the Cancer Registry of non-malignancies, or tumours which are not generally fatal, is acknowledged to be less complete (Nwene & Smith, 1982). Previous reports indicate that prostate cancer may be associated with breast cancer in families. Since carcinoma of the prostate, in general, is a late-onset cancer, we examined the incidence of carcinoma at this site for all ages. In all tables the totals are for cancers diagnosed at age less than 75, but for carcinoma of the prostate all patients were included regardless of age.

Observed numbers of cancer were compared with the expected, and a two-sided Poisson probability calculated. Relative risk ratios are estimated by dividing observed by expected numbers of cancers, and 95% confidence intervals calculated. All statistical analysis was done using Epilog Plus, version 2 (1987).

**Results**

The 402 interviewed patients led to 399 families, as there were two instances of sibling pairs and one instance of a niece–aunt pair within the patient set. There were a total of 2,867 first-degree relatives. Complete information (i.e. date of birth, age and status at last observation) was known on 2,630 of these, and 2,101 were at risk after 1 January 1965. Information was available on 4,405 second-degree relatives; of these 3,275 were at risk after 1 January 1965.

For first-degree relatives, during the 'at-risk' time period, 165 cancers (153 of which were confirmed) were experienced by 161 relatives. In 133 second-degree relatives a total of 136 cancers (107 of which were confirmed) were reported for the same 'at-risk' period.

Overall cancer risk in the relatives is presented in Table I. In the first-degree relatives there is a measurable excess for both sexes. The second-degree relatives have an excess, but this is not statistically significant. Table II shows the distribution of observed cancers by age group. Female first-degree relatives have a marked excess of cancers in the 30–44 age group. This is due to the excess of breast cancers. Male first-degree relatives have an excess of cancers diagnosed between ages 60 and 74. For second-degree relatives there is a significant excess of cancers between ages 45 and 59. If all cancers in patients under age 60 are considered, an overall excess is seen [obs = 47, exp = 34.55, RR = 1.36, 95% confidence interval (IC) 1.00–1.81, $P = 0.050$)]. This is present in both males and females, but does not reach statistical significance in these separate groups.

Table III shows that first-degree relatives not only have an excess of carcinomas, which is present in both males and females, but also have a significant excess of bone and soft-tissue sarcomas (all the sarcomas were confirmed from medical records). For individual sites the females have a marked excess of breast cancer (3 of the 45 breast cancers were unconfirmed, as the relative lived overseas). Considering the breast cancers in more detail, daughters have a higher risk of breast cancer than mothers and sisters, but these relative risks are not statistically significantly different from each other (mothers, obs = 13, exp = 4.66, RR = 2.79, 95% CI 1.49–4.77, $P = 0.002$; sisters, obs = 26, exp = 10.8, RR = 2.41, 95% CI 1.57–3.53, $P = 0.00012$; daughters, obs = 6, exp = 1.31, RR = 4.58, 95% CI 1.68–9.97, $P = 0.004$). However, if the female relatives are generally at a higher relative risk of early-onset breast cancer, then the possible raised risk in daughters could merely be a reflection of their younger ages. Males alone have a significant excess

**Table I** All cancers by sex and type of relative

| | *Number of relatives* | *Observed* | *Expected* | *Relative risk* | *95% CI* | *P-value* |
|---|---|---|---|---|---|---|
| *First-degree relatives* | | | | | | |
| Father | 192 | 31 | 22.58 | 1.37 | 0.93–1.95 | 0.11 |
| Brothers | 495 | 52 | 41.09 | 1.27 | 0.95–1.66 | 0.11 |
| Sons | 372 | 1 | 3.25 | 0.31 | 0.01–1.71 | 0.33 |
| Male | 1,059 | 84 | 66.92 | 1.26 | 1.00–1.56 | 0.047 |
| Mothers | 244 | 25 | 19.92 | 1.26 | 0.81–1.85 | 0.30 |
| Sisters | 447 | 49 | 38.26 | 1.28 | 0.95–1.69 | 0.091 |
| Daughters | 351 | 7 | 4.25 | 1.65 | 0.66–3.39 | 0.28 |
| Female | 1,042 | 81 | 62.43 | 1.30 | 1.04–1.63 | 0.022 |
| All | 2,101 | 165 | 129.35 | 1.28 | 1.09–1.49 | 0.002 |
| *Second-degree relatives* | | | | | | |
| Maternal male | 612 | 42 | 35.28 | 1.19 | 0.86–1.61 | 0.30 |
| Paternal male | 424 | 26 | 22.31 | 1.17 | 0.76–1.71 | 0.49 |
| Male | 1,614 | 73 | 64.55 | 1.13 | 0.89–1.42 | 0.32 |
| Maternal female | 706 | 31 | 28.21 | 1.10 | 0.75–1.56 | 0.65 |
| Paternal female | 473 | 24 | 18.81 | 1.28 | 0.82–1.90 | 0.28 |
| Female | 1,661 | 63 | 54.82 | 1.15 | 0.88–1.47 | 0.30 |
| All | 3,275 | 136 | 119.37 | 1.14 | 0.95–1.34 | 0.14 |

Observed, observed number of cancers; expected, expected number of cancers; relative risk, observed/expected; CI, confidence interval for the relative risk.

**Table II** All cancers by age at diagnosis of the relative

| *Age at diagnosis (years)* | *Obs* | *Exp* | *RR* | *95% CI* | *P-value* |
|---|---|---|---|---|---|
| *First-degree relatives* | | | | | |
| Under 15 | | | | | |
| Male | 1 | 0.31 | 3.21 | 0.08-17.88 | 0.54 |
| Female | 0 | 0.24 | – | 0.00-15.57 | 1.0 |
| | (0) | (0) | (–) | (–) | (–) |
| All | 1 | 0.55 | 1.82 | 0.05-10.15 | 0.84 |
| 15-29 | | | | | |
| Male | 1 | 1.06 | 0.94 | 0.02-5.26 | 1.00 |
| Female | 1 | 0.99 | 1.01 | 0.03-5.63 | 1.00 |
| | (1) | (0.11) | (9.39) | (10.23-50.65) | (0.20) |
| All | 2 | 2.05 | 0.98 | 0.12-3.53 | 1.00 |
| 30-44 | | | | | |
| Male | 2 | 3.36 | 0.60 | 0.07-2.15 | 0.70 |
| Female | 20 | 5.58 | 3.58 | 2.19-5.54 | <0.0001 |
| | (12) | (2.33) | (5.15) | (2.66-9.00) | (0.0001) |
| All | 22 | 8.94 | 2.46 | 1.54-3.73 | 0.00032 |
| 45-59 | | | | | |
| Male | 22 | 18.02 | 1.22 | 0.77-1.85 | 0.40 |
| Female | 20 | 19.98 | 1.00 | 0.61-1.55 | 1.0 |
| | (12) | (6.46) | (1.86) | (0.96-3.24) | (0.065) |
| All | 42 | 37.99 | 1.11 | 0.80-1.50 | 0.55 |
| 60-74 | | | | | |
| Male | 58 | 44.18 | 1.31 | 1.00-1.70 | 0.051 |
| Female | 40 | 35.64 | 1.12 | 0.81-1.55 | 0.46 |
| | (20) | (7.74) | (2.58) | (1.58-2.71) | (0.00033) |
| All | 98 | 79.82 | 1.23 | 1.00-1.51 | 0.046 |
| *Second-degree relatives* | | | | | |
| Under 15 | | | | | |
| Male | 2 | 0.69 | 2.91 | 0.35-10.50 | 0.49 |
| Female | 0 | 0.53 | – | 0.00-6.92 | 0.95 |
| | (0) | (0) | (–) | (–) | (–) |
| All | 2 | 1.22 | 1.64 | 0.20-5.92 | 0.69 |
| 15-29 | | | | | |
| Male | 1 | 1.54 | 0.65 | 0.02-3.62 | 1.00 |
| Female | 1 | 1.39 | 0.72 | 0.02-4.04 | 1.00 |
| | (0) | (0.13) | (–) | (0-28.38) | (1.00) |
| All | 2 | 2.93 | 0.68 | 0.08-2.46 | 0.88 |
| 30-44 | | | | | |
| Male | 2 | 2.33 | 0.86 | 0.10-3.10 | 1.00 |
| Female | 5 | 4.04 | 1.24 | 0.40-2.90 | 0.75 |
| | (3) | (1.63) | (1.84) | (0.38-5.38) | (0.45) |
| All | 7 | 6.37 | 1.10 | 0.44-2.27 | 0.90 |
| 45-59 | | | | | |
| Male | 18 | 11.60 | 1.55 | 0.92-2.45 | 0.10 |
| Female | 18 | 12.36 | 1.46 | 0.86-2.30 | 0.16 |
| | (8) | (3.89) | (2.06) | (0.89-4.05) | (0.090) |
| All | 36 | 24.03 | 1.50 | 1.05-2.08 | 0.03 |
| 60-74 | | | | | |
| Male | 50 | 48.12 | 1.04 | 0.77-1.37 | 0.82 |
| Female | 39 | 36.28 | 1.07 | 0.76-1.47 | 0.69 |
| | (10) | (8.00) | (1.25) | (0.60-2.30) | (0.57) |
| All | 89 | 84.40 | 1.05 | 0.85-1.30 | 0.65 |

Obs, observed number of cancers; Exp, expected number of cancers; RR, relative risk estimate, obs/exp; CI, confidence interval for the relative risk; numbers in brackets are breast cancers only.

of carcinoma of lip, oral cavity and pharynx (two of these were unconfirmed reports, but there is still a significant excess if these are excluded), a non-significant excess of carcinoma of the colon and rectum, and an excess of other and unspecified sites of borderline significance (seven of these were carcinomas of unknown or unspecified site; one was an unconfirmed report).

Table IV lists observed and expected numbers of cancers by morphological type in the second-degree relatives. There is a highly significant excess in the other and unspecified morphological group. The observed 17 cancers here include two neuroblastomas in young children; the remaining 15 are all 'cancer not otherwise specified', 10 of which are unconfirmed patient reports. There is still a significant excess in this category if the unconfirmed reports are ignored ($P = 0.0068$). Although overall no excess of carcinomas is seen, there is a borderline excess of breast carcinomas ($P = 0.07$, all confirmed) and a significant excess of kidney carcinoma (all confirmed) for females. For the males there is a significant deficit of carcinoma of the prostate and a significant excess of carcinoma of the trachea and lung (27 out of 34 confirmed). The excess of carcinoma of the lip, oral cavity and pharynx is not seen in the second-degree relatives, nor is the excess of sarcomas.

Table V analyses risk of cancers in the relatives by features in the index patient. Figures for breast cancer alone are given

**Table III** All cancers by morphological type in first-degree relatives

| | *Obs* | *Exp* | *RR* | *95% CI* | *P-value* |
|---|---|---|---|---|---|
| *Carcinoma* | | | | | |
| Male | 76 | 58.30 | 1.30 | 1.03– 1.63 | 0.029 |
| Female | 72 | 55.34 | 1.30 | 1.03– 1.65 | 0.030 |
| All | 148 | 113.64 | 1.30 | 1.12– 1.56 | 0.0018 |
| *Site of carcinoma* | | | | | |
| Bladder | | | | | |
| Male | 6 | 4.33 | 1.39 | 0.51– 3.02 | 0.53 |
| Female | 0 | 1.58 | – | 0.00– 2.35 | 0.42 |
| All | 6 | 5.91 | 1.02 | 0.37– 2.22 | 1.00 |
| Breast | | | | | |
| Male | 0 | 0.12 | – | 0.00–30.74 | 1.0 |
| Female | 45 | 16.77 | 2.68 | 1.97– 3.62 | <0.0001 |
| All | 45 | 16.89 | 2.66 | 1.96– 3.59 | <0.0001 |
| Cervix | | | | | |
| Female | 5 | 4.48 | 1.12 | 0.36– 2.62 | 0.92 |
| Colon and rectum | | | | | |
| Male | 13 | 7.99 | 1.63 | 0.87– 2.79 | 0.13 |
| Female | 4 | 7.37 | 0.54 | 0.15– 3.22 | 0.30 |
| All | 17 | 15.36 | 1.11 | 0.65– 1.78 | 0.72 |
| Endometrium | | | | | |
| Female | 1 | 1.87 | 0.53 | 0.01– 3.01 | 0.89 |
| Kidney | | | | | |
| Male | 0 | 1.28 | – | 0.00– 2.88 | 0.56 |
| Female | 1 | 0.76 | 1.32 | 0.03– 7.43 | 1.0 |
| All | 1 | 2.04 | 0.49 | 0.01– 2.74 | 0.79 |
| Larynx | | | | | |
| Male | 1 | 1.36 | 0.74 | 0.01– 4.10 | 1.0 |
| Female | 0 | 0.30 | – | 0.00–12.30 | 1.0 |
| All | 1 | 1.66 | 0.60 | 0.02– 3.36 | 1.0 |
| Lip, oral cavity and pharynx | | | | | |
| Male | 7 | 1.66 | 4.22 | 1.71– 8.74 | 0.0032 |
| Female | 0 | 0.86 | – | 0.00– 4.34 | 0.85 |
| All | 7 | 2.52 | 2.78 | 1.12– 5.75 | 0.03 |
| Ovary | | | | | |
| Female | 1 | 3.36 | 0.30 | 0.01– 1.67 | 0.31 |
| Pancreas | | | | | |
| Male | 4 | 2.17 | 1.84 | 0.50– 4.72 | 0.35 |
| Female | 2 | 1.60 | 1.25 | 0.15– 4.57 | 0.94 |
| All | 6 | 3.77 | 1.59 | 0.59– 3.48 | 0.35 |
| Prostate (all ages) | | | | | |
| Male | 9 | 8.15 | 1.10 | 0.51– 2.11 | 0.82 |
| Stomach | | | | | |
| Male | 9 | 5.81 | 1.55 | 0.71– 2.95 | 0.26 |
| Female | 3 | 3.17 | 0.95 | 0.20– 2.80 | 1.0 |
| All | 12 | 8.98 | 1.34 | 0.69– 2.35 | 0.38 |
| Trachea and lung | | | | | |
| Male | 21 | 23.42 | 0.90 | 0.56– 1.37 | 0.72 |
| Female | 4 | 6.88 | 0.58 | 0.16– 1.50 | 0.38 |
| All | 25 | 30.30 | 0.83 | 0.53– 1.22 | 0.40 |
| Uterus | | | | | |
| Female | 1 | 0.64 | 1.56 | 0.04– 8.84 | 0.94 |
| Other and unspecified | | | | | |
| Male | 11 | 6.26 | 1.76 | 0.88– 3.15 | 0.10 |
| Female | 5 | 5.70 | 0.88 | 0.29– 2.06 | 1.00 |
| All | 16 | 11.96 | 1.34 | 0.76– 2.17 | 0.58 |
| *CNS tumour* | | | | | |
| Male | 2 | 1.94 | 1.03 | 0.12– 3.72 | 1.0 |
| Female | 1 | 1.71 | 0.58 | 0.01– 3.28 | 0.99 |
| All | 3 | 3.65 | 0.82 | 0.17– 2.41 | 1.0 |
| *Leukaemia and lymphoma* | | | | | |
| Male | 4 | 4.21 | 0.95 | 0.26– 2.43 | 1.0 |
| Female | 4 | 3.16 | 1.27 | 0.35– 3.26 | 0.77 |
| All | 8 | 7.37 | 1.09 | 0.47– 2.15 | 0.90 |

Table III (cont.)

| | Obs | Exp | RR | 95% CI | P-value |
|---|---|---|---|---|---|
| *Bone and soft-tissue sarcoma* | | | | | |
| Male | 2 | 0.68 | 2.94 | 0.36–10.61 | 0.30 |
| Female | 4 | 0.72 | 5.52 | 1.51–14.23 | 0.013 |
| All | 6 | 1.41 | 4.26 | 1.57– 9.33 | 0.0064 |
| *Melanoma* | | | | | |
| Male | 0 | 0.54 | – | 0.00– 6.86 | 1.00 |
| Female | 0 | 0.94 | – | 0.00– 3.93 | 0.78 |
| All | 0 | 1.48 | – | 0.00– 2.50 | 0.46 |
| *Other and unspecified* | | | | | |
| Male | 0 | 1.25 | – | 0.00– 2.95 | 0.65 |
| Female | 0 | 0.55 | – | 0.00– 6.92 | 1.00 |
| All | 0 | 1.80 | – | 0.00– 2.04 | 0.35 |

Obs, observed number of cancers; Exp, expected number of cancers; RR, relative risk estimate, obs/exp; CI, confidence interval for RR.

Table IV All cancers by morphological type in second-degree relatives

| | Obs | Exp | RR | 95% CI | P-value |
|---|---|---|---|---|---|
| *Carcinoma* | | | | | |
| Male | 52 | 56.06 | 0.93 | 0.69– 1.22 | 0.65 |
| Female | 52 | 48.02 | 1.08 | 0.37– 1.42 | 0.60 |
| All | 104 | 104.08 | 1.00 | 0.82– 1.22 | 1.00 |
| *Site of carcinoma* | | | | | |
| Bladder | | | | | |
| Male | 1 | 4.04 | 0.25 | 0.01– 1.38 | 0.18 |
| Female | 2 | 1.40 | 1.43 | 0.17– 5.16 | 0.82 |
| All | 3 | 5.44 | 0.55 | 0.11– 1.61 | 0.42 |
| Breast | | | | | |
| Male | 0 | 0.11 | – | 0.00–33.54 | 1.00 |
| Female | 21 | 13.58 | 1.55 | 0.96– 2.36 | 0.07 |
| All | 21 | 13.69 | 1.53 | 0.95– 2.34 | 0.08 |
| Cervix | | | | | |
| Female | 3 | 3.73 | 0.80 | 0.17– 2.35 | 0.98 |
| Colon and rectum | | | | | |
| Male | 4 | 7.63 | 0.52 | 0.14– 1.34 | 0.25 |
| Female | 6 | 6.97 | 0.86 | 0.32– 1.87 | 0.91 |
| All | 10 | 14.60 | 0.69 | 0.33– 1.26 | 0.28 |
| Endometrium | | | | | |
| Female | 0 | 1.60 | – | 0.00– 2.30 | 0.40 |
| Kidney | | | | | |
| Male | 0 | 1.10 | – | 0.00– 3.35 | 0.67 |
| Female | 5 | 0.67 | 7.46 | 2.42–17.42 | 0.0012 |
| All | 5 | 1.76 | 2.84 | 0.92– 6.62 | 0.068 |
| Larynx | | | | | |
| Male | 0 | 1.19 | – | 0.00– 3.10 | 0.61 |
| Female | 0 | 0.24 | – | 0.00–15.37 | 1.00 |
| All | 0 | 1.43 | – | 0.00– 2.58 | 0.48 |
| Lip, oral cavity and pharynx | | | | | |
| Male | 1 | 1.47 | 0.68 | 0.02– 3.79 | 1.00 |
| Female | 0 | 0.78 | – | 0.00– 4.73 | 0.91 |
| All | 1 | 2.26 | 0.44 | 0.01– 2.47 | 0.68 |
| Ovary | | | | | |
| Female | 2 | 2.80 | 0.72 | 0.09– 2.58 | 0.94 |
| Pancreas | | | | | |
| Male | 1 | 2.09 | 0.48 | 0.01– 2.67 | 0.76 |
| Female | 1 | 1.53 | 0.65 | 0.02– 3.64 | 1.00 |
| All | 2 | 3.62 | 0.55 | 0.07– 1.99 | 0.60 |
| Prostate (all ages) | | | | | |
| Male | 3 | 9.84 | 0.30 | 0.06– 0.89 | 0.021 |
| Stomach | | | | | |
| Male | 8 | 5.95 | 1.34 | 0.58– 2.65 | 0.50 |
| Female | 1 | 3.29 | 0.30 | 0.01– 1.69 | 0.32 |
| All | 9 | 9.24 | 0.97 | 0.45– 1.85 | 1.00 |

**Table IV** (*cont.*)

| | *Obs* | *Exp* | *RR* | *95% CI* | *P-value* |
|---|---|---|---|---|---|
| Trachea and lung | | | | | |
| Male | 34 | 22.70 | 1.50 | 1.04– 2.09 | 0.032 |
| Female | 4 | 5.63 | 0.71 | 0.19– 1.82 | 0.68 |
| All | 38 | 28.32 | 1.34 | 0.95– 1.84 | 0.09 |
| Uterus | | | | | |
| Female | 0 | 0.54 | – | 0.00– 6.83 | 1.00 |
| Other and unspecified | | | | | |
| Male | 2 | 5.73 | 0.35 | 0.04– 1.26 | 0.14 |
| Female | 7 | 5.26 | 1.33 | 0.54– 2.74 | 0.54 |
| All | 9 | 11.00 | 0.82 | 0.37– 1.55 | 0.68 |
| *CNS tumour* | | | | | |
| Male | 3 | 1.79 | 1.76 | 0.35– 4.90 | 0.53 |
| Female | 2 | 1.58 | 1.27 | 0.15– 4.57 | 0.93 |
| All | 5 | 3.36 | 1.49 | 0.48– 3.47 | 0.50 |
| *Leukaemia and lymphoma* | | | | | |
| Male | 5 | 4.25 | 1.18 | 0.38– 2.75 | 0.84 |
| Female | 5 | 3.12 | 1.60 | 0.52– 3.74 | 0.41 |
| All | 10 | 7.37 | 1.36 | 0.65– 2.49 | 0.42 |
| *Bone and soft-tissue sarcoma* | | | | | |
| Male | 0 | 0.73 | – | 0.00– 5.06 | 0.96 |
| Female | 0 | 0.73 | – | 0.00– 5.03 | 0.96 |
| All | 0 | 1.46 | – | 0.00– 2.52 | 0.46 |
| *Melanoma* | | | | | |
| Male | 0 | 0.43 | – | 0.00– 8.55 | 1.0 |
| Female | 0 | 0.74 | – | 0.00– 5.01 | 0.96 |
| All | 0 | 1.17 | – | 0.00– 3.16 | 0.62 |
| *Other and unspecified* | | | | | |
| Male | 13 | 1.29 | 10.08 | 5.37–17.24 | <0.0001 |
| Female | 4 | 0.63 | 6.37 | 1.74–16.30 | 0.0004 |
| All | 17 | 1.92 | 8.85 | 5.16–14.18 | <0.0001 |

Obs, observed number of cancers; Exp, expected number of cancers; RR, relative risk estimate, obs/exp; CI, confidence interval for RR.

in brackets. A higher risk to relatives of bilateral breast cancer patients was not found in this series, and the risk was equivalent to that in relatives of patients with unilateral disease. For breast cancers alone, the risk to first-degree relatives of patients with bilateral disease is similar to the risk to first-degree relatives of patients with unilateral disease. In second-degree relatives the risk is higher if the patient has bilateral disease but the numbers in both groups are very small. When age at diagnosis of the proband is considered, both first- and second-degree relatives of patients diagnosed between 40 and 49 have a higher risk (although not statistically significantly higher) of cancers than relatives of patients diagnosed at other ages, but a significant excess of cancers in first-degree relatives of patients diagnosed at other ages is also found (obs = 128, exp = 106.77, RR = 1.20, 95% CI 1.11–1.45, $P = 0.040$). A similar pattern of risk emerges when breast cancers alone are considered. It is notable that the risk to relatives of patients diagnosed at less than 40 years did not differ from that in relatives of patients diagnosed over 50 years, although numbers were small in this group.

A further breakdown of breast cancers alone by age at diagnosis of the index patient is presented in Table VI. The breast cancer incidence is shown by age group of the relative. This table is of limited interest in itself as many of the cells are empty, but it may be useful to compare with results from other studies.

The more close relatives with breast cancer a woman has, the higher her own risk of breast cancer (Claus *et al.*, 1990). If we therefore look only at families in which the patient has at least one other first-degree relative with breast cancer, we would expect the relative risk of breast cancers to be proportionately higher than for all families. We might expect that the risk for other cancers would also be raised. To evaluate this hypothesis we analysed risks of cancers in those families in which the index patient has a first-degree female relative with breast cancer (Table VII). The relative risk here is lower than that for all families, although for males alone it is the same. For the second-degree relatives in these families (obs = 29, exp = 20.86, RR = 1.39, 95% CI 0.93–1.35, $P = 0.11$), the relative risk is higher but not statistically significant.

The analysis of the first-degree relatives' cancers gave us three distinct excess groups: sarcomas; carcinoma of breast and lip; and carcinomas of the oral cavity and pharynx. The cancer experience of second-degree relatives in families in which the patient has at least one first-degree relative with one of these cancers is summarised in Table VIII. There is a borderline excess in the female relatives, but overall the relative risk is unchanged. The individual sites that were in excess for the second-degree relatives as a whole were also examined. There is a higher risk of breast cancer in the relatives of a patient with a first-degree relative affected, but this is not statistically significant. The excess of carinoma of the kidney in the female relatives is equally spread between the two groups, as is the excess of trachea and lung carcinoma in males. This would suggest that the excesses of cancers (except for carcinoma breast) are not clustering in the same families.

**Table V** Distribution of cancers in the relatives, by features in the index patient

| | Number of index patients | First-degree relatives No. FDR | Obs | Exp | RR | 95% CI | P-value | Second-degree relatives No. SDR | Obs | Exp | RR | 95% CI | P-value |
|---|---|---|---|---|---|---|---|---|---|---|---|---|---|
| *Index laterality* | | | | | | | | | | | | | |
| Right | 183 | 976 | 81 (20) | 60.88 (8.22) | 1.33 (2.43) | 1.06–1.65 (1.49–3.76) | 0.014 (0.00058) | 1511 | 61 (8) | 50.93 (5.63) | 1.20 (1.42) | 0.92–1.54 (0.61–2.80) | 0.18 (0.41) |
| Left | 197 | 1015 | 75 (22) | 60.25 (7.61) | 1.25 (2.89) | 0.98–1.56 (1.81–4.38) | 0.073 (0.00003) | 1582 | 69 (11) | 61.68 (7.24) | 1.12 (1.52) | 0.87–1.42 (0.76–2.72) | 0.38 (0.23) |
| Bilateral | 22 | 110 | 9 (3) | 8.22 (1.06) | 1.09 (2.83) | 0.50–2.08 (0.58–8.27) | 0.88 (0.18) | 182 | 6 (2) | 6.76 (0.81) | 0.89 (2.47) | 0.33–1.93 (0.30–8.92) | 0.97 (0.39) |
| *Index age at diagnosis* | | | | | | | | | | | | | |
| Less than 40 | 32 | 194 | 10 (3) | 9.09 (1.11) | 1.10 (2.70) | 0.53–2.02 (0.56–7.90) | 0.85 (0.20) | 270 | 17 (1) | 16.91 (1.95) | 1.01 (0.51) | 0.59–1.61 (0.01–2.86) | 1.0 (0.84) |
| 40–49 | 74 | 489 | 37 (14) | 22.59 (2.97) | 1.64 (4.71) | 1.15–2.26 (2.58–7.91) | 0.007 (0.00001) | 739 | 49 (10) | 34.15 (3.98) | 1.43 (2.51) | 1.06–1.90 (1.20–4.62) | 0.020 (0.02) |
| 50–59 | 110 | 595 | 43 (11) | 35.39 (4.87) | 1.22 (2.26) | 0.88–1.64 (1.13–4.04) | 0.24 (0.02) | 1025 | 40 (5) | 39.56 (4.18) | 1.01 (1.20) | 0.72–1.38 (0.39–2.79) | 0.99 (0.81) |
| 60+ | 186 | 823 | 75 (17) | 62.29 (7.93) | 1.20 (2.14) | 0.96–1.52 (1.25–3.43) | 0.11 (0.0058) | 1241 | 30 (5) | 28.74 (3.58) | 1.04 (1.40) | 0.70–1.49 (0.45–3.26) | 0.86 (0.58) |

No. FDR, number of first-degree relatives; No. SDR, number of second-degree relatives; Obs, observed number of cancers; Exp, expected number of cancers; RR, relative risk estimate, obs/exp; CI, confidence interval for the relative risk; numbers in brackets are for carcinoma of the breast only.

**Table VI** Breast cancers by age of index patient and age of relative

| Age group | Age of index patient at diagnosis: Under 40 | 40–49 | 50–59 | 60 plus |
|---|---|---|---|---|
| *First-degree relative* | | | | |
| Under 40 | | | | |
| O | 0 | 6 | 2 | 3 |
| E | 0.083 | 0.26 | 0.34 | 0.46 |
| *P* | 1.0 | <0.0001 | 0.092 | 0.022 |
| 40–49 | | | | |
| O | 0 | 2 | 1 | 0 |
| E | 0.25 | 0.67 | 1.16 | 1.27 |
| *P* | 1.0 | 0.29 | 1.0 | 0.56 |
| 50–59 | | | | |
| O | 2 | 0 | 4 | 5 |
| E | 0.35 | 0.75 | 1.32 | 2.02 |
| *P* | 0.099 | 0.95 | 0.091 | 0.11 |
| 60–74 | | | | |
| O | 1 | 6 | 4 | 9 |
| E | 0.42 | 1.30 | 2.02 | 4.11 |
| *P* | 0.68 | 0.0045 | 0.29 | 0.049 |
| *Second-degree relative* | | | | |
| Under 40 | | | | |
| O | 0 | 0 | 0 | 2 |
| E | 0.046 | 0.060 | 0.23 | 0.64 |
| *P* | 1.0 | 1.0 | 1.0 | 0.27 |
| 40–49 | | | | |
| O | 1 | 1 | 0 | 2 |
| E | 0.32 | 0.36 | 0.20 | 0.94 |
| *P* | 0.55 | 0.61 | 1.0 | 0.49 |
| 50–59 | | | | |
| O | 0 | 4 | 0 | 1 |
| E | 0.56 | 1.10 | 0.70 | 0.49 |
| *P* | 1.0 | 0.052 | 0.99 | 0.77 |
| 60–74 | | | | |
| O | 0 | 5 | 5 | 0 |
| E | 1.00 | 2.42 | 3.02 | 1.48 |
| *P* | 0.73 | 0.20 | 0.37 | 0.46 |

O, observed number of cancers; E, expected number of cancers; *P*, *P*-value.

### Discussion

The breast cancer probands in this series included all patients treated with surgery at particular hospitals over a specific time period, and are therefore not subject to the selection biases present in other series based on early age at diagnosis or family history. There is a measurable excess risk of all incident cancers to both male and female first-degree relatives and a non-significant excess in the second-degree relatives. Anderson *et al.* (1992) also found a similar, but non-significant, result when looking at cancer deaths in first-degree relatives of young breast cancer patients. The excess of cancers in the females is almost completely due to breast cancers, but there is also a significant excess of bone and soft-tissue sarcomas in the first-degree relatives and an excess of carcinoma of the kidney in the second-degree female cohort. Male first-degree relatives are at increased risk of carcinomas (of any or unspecified site), and the specific sites which are in excess differ in first- and second-degree relatives.

The excess of cancers observed in second-degree relatives is lower than in first-degree relatives. Applying the argument of Risch (1990), assuming that breast cancer is the result of a single susceptibility locus and the relative risk in first-degree relatives is 1.28, we would expect a risk in second-degree relatives of 1.14. This is in fact what we see, and the same relationship holds true for males and females examined separately. Following the same argument, we would expect the relative risk of breast cancer in second-degree relatives to

**Table VII** Distribution of all cancers (and breast cancers) in the first-degree relatives of cases with a mother, sister or daughter with breast cancer[a]

| *Relationship* | *Number of index patients* | *Number of first-degree relatives* | *Observed* | *Expected* | *Relative risk* | *95% CI* | *P-value* |
|---|---|---|---|---|---|---|---|
| All | 63 | 383 | 22 | 20.32 | 1.08 | 0.68-1.64 | 0.71 |
| | | | (5) | (2.64) | (1.89) | (0.61-4.42) | (0.25) |
| Male | | 206 | 14 | 10.58 | 1.32 | 0.72-2.22 | 0.35 |
| | | | (0) | (0.01) | (–) | (–) | (1.00) |
| Female | | 177 | 8 | 9.74 | 0.82 | 0.35-1.62 | 0.78 |
| | | | (5) | (2.63) | (1.90) | (0.62-4.44) | (0.25) |
| Fathers | | 62 | 7 | 4.07 | 1.72 | 0.69-3.54 | 0.24 |
| | | | (0) | (0.00) | (–) | (–) | (1.0) |
| Brothers | | 90 | 7 | 6.13 | 1.14 | 0.46-2.35 | 0.83 |
| | | | (0) | (0.01) | (–) | (–) | (1.0) |
| Sons | | 54 | 0 | 0.38 | – | 0.00-9.71 | 1.00 |
| | | | (0) | (0.00) | (–) | (–) | (1.0) |
| Mothers | | 29 | 0 | 1.32 | – | 0.00-2.79 | 0.54 |
| | | | (0) | (0.30) | (–) | (0.00-12.30) | (1.00) |
| Sisters | | 92 | 8 | 7.79 | 1.03 | 0.44-2.02 | 1.00 |
| | | | (5) | (2.13) | (2.35) | (0.76-5.48) | (0.13) |
| Daughters | | 54 | 0 | 0.63 | – | 0.00-5.86 | 1.00 |
| | | | (0) | (0.20) | (–) | (0.00-18.44) | (1.00) |

[a]The first relative to be diagnosed with a breast cancer was removed from the cohort. Observed, observed number of cancers; Expected, expected number of cancers; Relative risk, observed/expected; CI, confidence interval for relative risk; figures in brackets are for breast cancers only.

be 1.84. This is not so different from the observed 1.55. However, all these observations are also consistent with a polygenic model.

For all cancers the relative risk in first-degree relatives is raised for all ages of index patient at diagnosis, but the risk is markedly higher in relatives of patients diagnosed in the age range 40-49. This age effect is also demonstrated in the second-degree relatives.

We have found no evidence that relatives of women diagnosed with bilateral breast cancer are at increased risk of breast cancer or cancer in general compared with relatives of those women with unilateral breast cancer. This could be because of the small number of patients with bilateral disease, but other studies have found bilaterality not to be a risk factor (Adami *et al.*, 1981; Claus *et al.*, 1990). It may also have been due, in part, to our rigid definition of a bilateral breast cancer patient. However, the status of 'bilateral' can only be a retrospective one. Some of the women who were unilateral when eligible for our study may subsequently develop another primary breast cancer. The long term follow-up of these women and their relatives may ultimately resolve this matter.

Tulinius *et al.* (1992) found an excess of prostate, endometrial and ovarian cancer in the relatives of women with breast cancer. They had specifically examined these sites, and the risks of cancers of other sites and histological groups were not reported. Arason *et al.* (1993) found that prostate cancer was frequently seen in breast cancer families selected for linkage analyses. In first-degree relatives of our breast cancer probands we did not see an excess of these cancers. However, this analysis included only those cancers diagnosed after 1964, and if cancers occurring before 1965 are included we observed 11 prostate carcinomas, five ovarian cancers (including one germ cell tumour) and two endometrial carcinomas. Out of these 18 families, there were eight with additional relatives with breast cancer. Four out of the five ovarian cancers, but only 1 of the 11 prostate cancers, appeared in patterns consistent with a dominant mode of inheritance. This study has sufficient statistical power to detect minimum relative risks of 1.84 for prostate carcinoma, 2.38 for ovarian carcinoma and of 3.20 for endometrial carcinoma in the first-degree cohort. These 'detectable' relative risks are all higher than those found by Tulinius *et al.* (1992). Our study, therefore, lacks the power to confirm, or refute, a relative risk of this magnitude, and it may be that in a small number of families these cancers do tend to cluster as a result of common aetiological factors.

There is an established excess risk of breast cancer for the mothers of children with a sarcoma (Li *et al.*, 1969; Birch *et al.*, 1984, 1990; Strong *et al.*, 1987). Bürki *et al.* (1987) found an excess of sarcomas in the brothers of breast cancer patients. Familial aggregations of sarcoma, breast cancer and other neoplasms at young ages is referred to as the Li-Fraumeni syndrome. The excess of sarcoma seen in relatives of women with breast cancer in the present series is compatible with these previous observations. Recently, mutations in the tumour-suppressor gene, p53, have been found in some individuals whose families exhibit the Li-Fraumeni syndrome (Malkin *et al.*, 1990; Santibáñez-Koref *et al.*, 1991). Among the second-degree relatives of our patients there were two children with neuroblastomas. It has recently been reported that a germline p53 mutation has been found in a patient diagnosed with a neuroblastoma at age 1 year who subsequently developed breast cancer at age 32 years. Her mother also had breast cancer (Malkin *et al.*, 1992). Furthermore, among series of breast cancer patients examined for p53 germline mutations, four examples have been found, all associated with a family history of breast and other cancers (Børreson *et al.*, 1992; Prosser *et al.*, 1992; Sidransky *et al.*, 1992). This is further evidence for breast cancer being a heterogeneous disease, and the 'Li-Fraumeni' component is actually measurable from a series such as ours.

The excess of sarcoma in first-degree relatives is not seen in the second-degree relatives, but there a number of cancers that could not be allocated to specific morphological groups because of imprecise information. The lack of an excess of particular types or sites here is not necessarily informative. Other studies may have failed to detect an excess of sarcoma because of inability to identify and calculate expected numbers in this morphological group. Most cancer registries classify patients according to the International Classification of Diseases (ICD), which groups cancers by the site of the tumour. ICD-O allows coding by specific histological type as well as the site of the tumour, and we have access to incidence rates based on ICD-O-coded cancer registry data for the north-west of England.

The tables presented list around 250 statistical tests. Although these are not all independent, we would expect

**Table VIII** Distribution of cancers in the second-degree relatives, by family with at least one first-degree relative of index with carcinoma of breast, lip, oral cavity or pharynx, or a sarcoma

| | First-degree relative not affected | | | | | | First-degree relative affected | | | | | |
|---|---|---|---|---|---|---|---|---|---|---|---|---|
| | No. SDR | Obs | Exp | RR | 95% CI | P-value | No. SDR | Obs | Exp | RR | 95% CI | P-value |
| *All cancers* | | | | | | | | | | | | |
| Male | 1258 | 59 | 49.65 | 1.19 | 0.90–1.53 | 0.21 | 356 | 14 | 14.90 | 0.94 | 0.51–1.58 | 1.00 |
| Female | 1272 | 42 | 41.77 | 1.01 | 0.72–1.36 | 1.00 | 389 | 21 | 13.05 | 1.61 | 1.00–2.46 | 0.051 |
| All | 2530 | 101 | 91.43 | 1.10 | 0.89–1.33 | 0.34 | 745 | 35 | 27.94 | 1.25 | 0.87–1.74 | 0.22 |
| *Carcinoma* | | | | | | | | | | | | |
| Breast | | | | | | | | | | | | |
| Male | | 0 | 0.09 | – | 0.00–40.99 | 1.0 | | 0 | 0.02 | – | 0.00–184.44 | 1.00 |
| Female | | 14 | 10.34 | 1.35 | 0.74–2.27 | 0.32 | | 7 | 3.23 | 2.17 | 0.87–4.47 | 0.094 |
| All | | 14 | 10.43 | 1.34 | 0.73–2.25 | 0.34 | | 7 | 3.25 | 2.15 | 0.87–4.44 | 0.097 |
| *Kidney* | | | | | | | | | | | | |
| Male | | 0 | 0.84 | – | 0.00–4.39 | 0.86 | | 0 | 0.26 | – | 0.00–14.19 | 1.00 |
| Female | | 3 | 0.51 | 5.91 | 1.22–17.27 | 0.030 | | 2 | 0.16 | 12.67 | 1.51–45.15 | 0.022 |
| All | | 3 | 1.35 | 2.22 | 0.46–6.49 | 0.31 | | 2 | 0.42 | 4.76 | 0.58–17.20 | 0.13 |
| *Trachea and lung* | | | | | | | | | | | | |
| Male | | 26 | 17.47 | 1.49 | 0.97–2.18 | 0.07 | | 8 | 5.23 | 1.53 | 0.66–3.01 | 0.17 |
| Female | | 2 | 4.29 | 0.47 | 0.06–1.68 | 0.40 | | 2 | 1.33 | 1.50 | 0.18–5.43 | 0.77 |
| All | | 28 | 21.76 | 1.29 | 0.86–1.86 | 0.22 | | 10 | 6.56 | 1.52 | 0.73–2.80 | 0.14 |

No. SDR, number of second-degree relatives; Obs, observed number of cancers; Exp, expected number of cancers; RR, relative risk estimate, obs/exp; CI, confidence interval for the relative risk.

12–13 tests to be significant at the 5% level and 2–3 to be significant at the 1% level, and these results would be due to chance alone. In our analysis we see considerably more than this, so although some of our significant results may be due to chance, they are not simply an artifact of repeated testing.

Recent developments in breast cancer linkage studies have demonstrated strong evidence of tight linkage to the region 17q21 (Hall *et al.*, 1990). The families that appeared to be linked to this site were large, with predominantly early age at onset. Easton *et al.* (1993) examined 214 breast cancer families for linkage to 17q21 and, although all breast-ovarian cancer families are consistent with linkage, there was significant evidence of genetic heterogeneity amongst the families for linkage to 17q21 and, although all breast–ovarian cancer families are consistent with linkage, there was this tightly linked gene to be very high (82% by age 70). If the gene responsible for familial breast cancer is highly penetrant and can also predispose to other cancers, then, by targeting those families with at least two first-degree relatives with breast cancer, a much higher incidence of breast and other cancers would be expected in this group. This is not demonstrated by our analysis, and the present data would support the hypothesis that a large proportion of breast cancer could be caused either by shared environmental factors within families, giving the relatives a higher than expected risk of cancer, or by a relatively common predisposing gene which has a low penetrance. A combination, or interaction, of genetic and environmental factors could also have a role.

Some families from our series were examined for linkage to 17q21, but the results were inconclusive (Teare *et al.*, 1993). The study by Skolnick *et al.* (1990) found evidence for a common breast cancer gene, with low penetrance, responsible for a considerable proportion of breast cancer. This is in contrast to the majority of segregation analyses, which have found familial clustering best described by a dominant rare gene with high penetrance (Newman *et al.*, 1988; Claus *et al.*, 1992; Iselius *et al.*, 1992).

The present study has found that relatives of breast cancer patients are at a higher risk of a variety of neoplasms than the general population. This excess of cancers, including breast cancer, is not limited to a small number of high-risk families but appears to be spread across many. Given the evidence for genetic heterogeneity which has now come to light convincingly through linkage analyses, the various different single-gene models found by segregation analysis to 'best fit' familial breast cancer could be explained by ascertainment bias, with each analysis correctly modelling the data collected. Breast cancer probands are very often selected for age at diagnosis or bilaterality. This could lead to an overascertainment of a particular subtype of familial breast cancer. Although our series is smaller than some other studies, it is nevertheless substantial and unselected. Furthermore, an unusually high proportion of cancers in relatives has been verified and fully documented, and many of the breast cancers have been subjected to special histopathological review. We are, therefore, in a good position to model patterns of inheritance of breast cancer and to examine the possibility that the excess of cancers at sites other than breast segregate with a predisposing gene. Segregation analyses on this data set are currently under way to clarify this issue.

We are most grateful to Professor R.A. Sellwood for his support with the project. Jillian M. Birch is a Cancer Research Campaign Reader in Oncology. This research was supported by the Cancer Research Campaign.

### References

ADAMI, H.O., HANSEN, J., YUNG, B. & RIMSTEN, A.J. (1981). Characteristics of familial breast cancer in Sweden. Absence of relation to age and unilateral and bilateral disease. *Cancer*, **48**, 1688–1695.

ANDERSON, D.E. (1971). Some characteristics of familial breast cancer. *Cancer*, **28**, 1500–1504.

ANDERSON, D.E. (1974). Genetic study of breast cancer: Identification of a high risk group. *Cancer*, **34**, 1090–1097.

ANDERSON, D.E. & BADZIOCH, M.D. (1985). Risk of familial breast cancer. *Cancer*, **56**, 383-387.

ANDERSON, K.E., EASTON, D.F., MATTHEWS, F.E. & PETO, J. (1992). Cancer mortality in the first degree relatives of young breast cancer patients. *Br. J. Cancer*, **66**, 599-602.

ANDRIEU, N., CLAVEL, F. & DEMENAIS, F. (1989). Familial susceptibility to breast cancer: a complex inheritance. *Int. J. Cancer*, **44**, 415-418.

ARASON, A., BARKARDÓTTIR, R.B. & EGILSSON, V. (1993). Linkage analysis of chromosome 17q markers and breast-ovarian cancer in Icelandic families, and possible relationship to prostatic cancer. *Am. J. Hum. Genet.*, **52**, 711-717.

BIRCH, J.M., HARTLEY, A.L., BLAIR, V., KELSEY, A.M., HARRIS, M., TEARE, M.D. & MORRIS-JONES, P.H. (1990). Cancer in the families of children with soft tissue sarcoma. *Cancer*, **66**, 2239-2248.

BIRCH, J.M., HARTLEY, A.L., MARSDEN, H.B., HARRIS, M. & SWINDELL, R. (1984). Excess risk of breast cancer in the mothers of children with soft tissue sarcomas. *Br. J. Cancer*, **49**, 325-331.

BØRRESEN, A.-L., ANDERSEN, T.I., GARBER, J., BARBIER-PIRAUX, N., THORLACIUS, S., EYFJÖRD, J., OTTESTAD, L., SMITH-SØRESEN, B., HOVIG, E., MALKIN, D. & FRIEND, S.H. (1992). Screening for germ line *TP*53 mutations in breast cancer patients. *Cancer Res.*, **52**, 3234-3236.

BÜRUKI, N., GENCIK, A., TORHORST, J.K.H., WEBER, W. & MÜLLER, H. (1987). Familial and histological analyses of 138 breast cancer patients. *Br. Cancer Res. Treat.*, **10**, 159-167.

CLAUS, E.B., RISCH, N.J. & THOMPSON, W.D. (1990). Age at onset as an indicator of familial risk of breast cancer. *Am. J. Epidemiol.*, **131**, 961-972.

CLAUS, E.B., RISCH, N.J. & THOMPSON, W.D. (1991). Genetic analysis of breast cancer in the Cancer and Steroid Hormone Study. *Am. J. Hum. Genet.*, **48**, 232-242.

EASTON, D.F., BISHOP, D.T., FORD, D. & CROCKFORD, G.P. (1993). Genetic linkage analysis in familial breast and ovarian cancer - results from 214 families. *Am. J. Hum. Genet.*, **52**, 670-678.

EPILOG PLUS (1987). *Epicenter Software*. Epicenter Software, Pasadena.

HALL, J.M., MING, K., NEWMAN, B., MORROW, J.E., ANDERSON, L.A., HUEY, B. & KING, M.-C. (1990). Linkage of early-onset breast cancer to chromosome 17q21. *Science*, **250**, 1684-1689.

HALL, J.M., FRIEDMAN, L., GUENTHER, C., LEE, M.K., WEBER, J.L., BLACK, D.M., KING, M.-C. (1992). Closing in on a breast cancer gene on chromosome 17q. *Am. J. Hum. Genet.*, **50**, 1235-1242.

ISELIUS, L., LITTLER, M. & MORTON, N. (1992). Transmission of breast cancer - a controversy resolved. *Clin. Genet.*, **41**, 211-217.

LI, F.P., FRAUMENI, Jr, J.R. (1969). Rhabdomyosarcoma in children: Epidemiologic study and identification of a familial cancer syndrome. *J. Natl Cancer Inst.*, **43**, 1365-1373.

MALKIN, D., LI, F.P., STRONG, L.C., FRAUMENI, Jr, J.F., NELSON, C.E., KIM, D.H., KASSEL, J., GRYKA, M.G., BISCHOFF, F.Z., TAINSKY, M.A. & FRIEND, S.H. (1990). Germline p53 mutations in a familial syndrome of breast cancer, sarcomas and other neoplasms. *Science*, **250**, 1233-1238.

MALKIN, D., JOLLY, K.W., BARBIER, N., LOOK, A.T., FRIEND, S.H., GEBHARDT, M.C., ANDERSEN, T.I., BØRRESEN, A.-L., LI, F.P., GARBER, J. & STRONG, L.C. (1992). Germline mutations of the p53 tumor-suppressor gene in children and young adults with second malignant neoplasms. *N. Engl. J. Med.*, **326**, 1309-1315.

NEWMAN, B., AUSTIN, M.A., LEE, M. & KING, M.-C. (1988). Inheritance of human breast cancer: evidence for autosomal dominant transmission in high-risk families. *Proc. Natl Acad. Sci. USA*, **85**, 3044-3048.

NWENE, U. & SMITH, A. (1982). Assessing completeness of cancer registration in the North-Western region of England by a method of independent comparison. *Br. J. Cancer*, **46**, 635-639.

PARKIN, D.M., LÄÄRA, E. & MUIR, C.S. (1988). Estimates of the worldwide frequency of sixteen major cancers in 1980. *Int. J. Cancer*, **41**, 184-197.

PROSSER, J., PORTER, D., COLES, C., CONDIE, A., THOMPSON, A.M., CHETTY, U., STEEL, C.M. & EVANS, H.J. (1992). Constitutional p53 mutation in a non-Li-Fraumeni cancer family. *Br. J. Cancer*, **65**, 527-528.

RISCH, N. (1990). Linkage strategies for genetically complex traits. I. Multilocus models. *Am. J. Hum. Genet.*, **46**, 222-228.

SANTIBÁÑEZ-KOREF, M.F., BIRCH, J.M., HARTLEY, A.L., MORRIS-JONES, P.H., CRAFT, A.H., EDEN, T., CROWTHER, D., KELSEY, A.M. & HARRIS, M. (1991). p53 germline mutations in Li-Fraumeni syndrome. *Lancet*, **338**, 1490-1491.

SIDRANSKY, D., TOKINO, T., HELZLSOUER, K., ZEHNBAUER, B., RAUSCH, G., SHELTON, B., PRESTIGIACOMO, L., VOGELSTEIN, B. & DAVIDSON, N. (1992). Inherited p53 gene mutations in breast cancer. *Cancer Res.*, **52**, 2984-2986.

SKOLNICK, M.H., CANNON-ALBRIGHT, L.A., GOLDGAR, D.E., WARD, J.H., MARSHALL, C.J., SCHUMANN, G.B., HOGLE, H., MCWHORTER, W.P., WRIGHT, E.C., TRAN, T.D., BISHOP, D.T., KUSHNER, J.P. & EYRE, H.J. (1990). Inheritance of proliferative breast disease in breast cancer kindreds. *Science*, **250**, 1715-1720.

STRONG, L.C., STINE, M. & NORSTED, T.L. (1987). Cancer in survivors of childhood soft tissue sarcoma and their relatives. *J. Natl Cancer Inst.*, **79**, 1213-1220.

TEARE, M.D., SANTIBÁÑEZ-KOREF, M.F., WALLACE, S.A., WHITE, G.R.M., EVANS, D.G.R., BURNELL, L.D., HARRIS, M., HOWELL, A. & BIRCH, J.M. (1993). A linkage study in seven breast cancer families. *Am. J. Hum. Genet.*, **52**, 786-788.

TULINIUS, H., EGILSSON, V., ÓLAFSDÓTTIR, G.H. & SIGVALDASON, H. (1992). Risk of prostate, ovarian, and endometrial cancer among relatives of women with breast cancer. *Br. Med. J.*, **305**, 855-857.

WILLIAMS, W.R. & ANDERSON, D.E. (1984). Genetic epidemiology of breast cancer: segregation analysis of 200 Danish pedigrees. *Genet. Epidemiol.*, **1**, 7-20.

WORLD HEALTH ORGANISATION (1976). ICD-O: *International Classification of Diseases for Oncology*. World Health Organization: Geneva.